# Effect of Copper, Zinc, and Selenium on the Migration of Bovine Neutrophils

**DOI:** 10.3390/vetsci8110281

**Published:** 2021-11-19

**Authors:** Hai Wang, Guanxin Lv, Shuai Lian, Jianfa Wang, Rui Wu

**Affiliations:** 1 College of Animal Science and Veterinary Medicine, Heilongjiang Bayi Agricultural University, Daqing 163319, China; whdota@foxmail.com (H.W.); lvarron@foxmail.com (G.L.); lianlianshuai@163.com (S.L.); wjflw@sina.com (J.W.); 2 Heilongjiang Provincial Key Laboratory of Prevention and Control of Bovine Diseases, Daqing 163319, China

**Keywords:** dairy cow, neutrophil, migration, trace elements

## Abstract

Neutrophils represent the first line of mammary gland defense against invading pathogens by transmigration across the mammary epithelial cell barrier. The effect of trace elements on the migration of bovine neutrophils is not clear. In this study, we investigated the effect of copper (Cu; 0.5, 1.0 and 1.5 mg/L), zinc (Zn; 1.0, 5.0 and 10 mg/L) and selenium (Se; 0.1, 1.0 and 2.0 mg/L) on the migration of bovine neutrophils by using a Transwell assay. The results showed that Cu, Zn and Se promoted the number of neutrophils in the trans-mammary epithelium. With the increased concentration of Cu at 1.5 mg/L, the number of neutrophils in the trans-mammary epithelium was increased significantly (*p* < 0.05). Zn (5.0 mg/L) and Se (0.1 mg/L) increased the migrated number of neutrophils (*p* < 0.01) to an extremely significant degree. These findings provided a theoretical and experimental basis for mammary gland immunity in dairy cows. Thus, we suggest that adding moderate amounts of different trace elements can improve the immune function of dairy cows.

## 1. Introduction

Neutrophils are the main leukocyte cells and play an important role in the non-specific immune system of dairy cows. In the healthy mammary gland, neutrophils have a low concentration of cell population (from 5 to 20%). When pathogens invade the bovine mammary gland, neutrophils are recruited to the mammary gland and execute important functions including phagocytosis, respiratory bursts, and the formation of an extracellular trapping network (NET) to kill pathogens [1,2,3,4]. The recruitment of neutrophils is a complex process including the following steps: tethering, rolling, adhesion, crawling, and trans-endothelial migration [5,6]. However, in the process of neutrophils performing their sterilization function, the reactive oxygen species (ROS), NETs and other cytotoxic effect molecules released by neutrophils will not only kill pathogens but also cause damage to the mammary gland itself [7,8]. It can be seen that neutrophils have a “double-edged sword” effect on the occurrence and development of dairy cows’ mastitis. Several studies have shown that the promptness and magnitude of the initial recruitment of neutrophils by the infected mammary gland have a profound influence on the severity and outcome of mastitis [9,10,11]. That is, when the mammary gland is infected, neutrophils need to immediately migrate from the bloodstream to the site of infection to kill pathogens.

Several researchers have found an association between trace elements in bovine nutrition and the ability to resist infections [12,13,14]. Several studies have identified that cattle during the periparturient period have a depression in blood levels of copper (Cu), zinc (Zn), and selenium (Se). These nutrients are components of metal chelates, metalloproteins and various reductases. For example, Cu and Zn are components of Cu/Zn-superoxide dismutase (Cu/Zn-SOD) against ROS [15]. Zn is essential and exists in almost all enzymes such as transferases, hydrolases, isomerases, oxidoreductases, and so on [16]. Similarly, many selenoprotein enzymes are related to immune function such as glutathione peroxidases (GPXs), thioredoxin reductases (TXNRDs), iodothyronine deiodinases (DIOs), and so on [17]. Since the 1980s, researchers have begun to study the effects of trace element deficiency on the function of neutrophils in animals. Boyne and Arthur found that neutrophils isolated from cattle fed Se, Cu, and Se/Cu-deficient diets were not affected the ability of the neutrophils to ingest *C. albicans*, but their ability to kill the ingested *C. albicans* was decreased [18]. Jones et al. and Olkowski et al. have similar study reports on sheep with Cu deficiency [19,20]. Percival et al. found that a lack of Cu can cause neutrophils reduction syndrome, which is manifested as a decrease in the number of neutrophils circulating in peripheral blood [21]. The transition metal Zn is an essential trace element indispensable for a suitable and sufficient immune response against pathogens. Studies have shown that Zn deficiency can affect neutrophils’ function and reduce the chemotaxis of human neutrophils [22]. Bednarek et al. found adding ZnSO_4_ to the diet of calves can increase Zn levels in the liver and serum, enhance macrophages’ random migration and change the proportion of neutrophils in peripheral blood [23]. Release of ROS is one of the ways that neutrophils kill invading pathogens. The content of ROS released and the ability to kill invading pathogens were significantly reduced in dairy cows with Se deficiency [24]. Supplementation of Se can effectively improve the chemotaxis and peroxide content of neutrophils [25]. Ndiweni and Finch found that neutrophils isolated from the blood of cows fed high levels of Se diet had a stronger ability to kill pathogens [26].

These studies suggest that the nutritional status of trace elements in dairy cows has an impact on the function of neutrophils. However, how trace elements Cu, Zn and Se affect the migration of bovine neutrophils is unknown. Therefore, the objective of this work is to investigate the effect of Cu, Zn and Se on the migration of neutrophils and provide useful information for the further study of trace elements on the function of neutrophils.

## 2. Materials and Methods

### 2.1. Animals, Blood Collection and Neutrophil Isolation

The test animals were selected from a sizeable intensive dairy farm in Daqing, Heilongjiang Province, China. Three healthy 2-year-old cows of similar body condition and at mid-lactation were randomly selected. All cows were healthy and did not exhibit any clinical signs of mastitis. Whole blood was collected from the cows via the middle tail vein. Neutrophils were isolated and purified by using the bovine peripheral blood neutrophil purification kit^®^ (Solarbio, Beijing, China). Then, 4 mL Separate A, 2 mL Separate C, and 2 mL sodium citrate anticoagulated whole blood were successively added into a 15 mL centrifuge tube, forming a liquid gradient. The mixture was then centrifuged at room temperature in an Eppendorf high-speed centrifuge at 900× *g* for 30 min. After the centrifugation step, neutrophils were found in the lower gradient phase as a white ring. Neutrophils were extracted into a new 15 mL centrifuge tube and erythrocytes were lysed with Red Blood Cell Lysis Buffer. Then, the suspensions were centrifuged (300× *g*, 5 min) and washed three times; neutrophils were resuspended in Roswell Park Memorial Institute (RPMI) 1640 medium (Gibco, Grand Island, NY, USA) containing 10% fetal bovine serum (FBS; Gibco Grand Island, NY, USA).

### 2.2. Detection of Neutrophil Survival

Separated neutrophils (2 μL) were used to make a Giemsa-stained smear, which was checked for purity by a Nikon Eclipse TS100 inverted fluorescence microscope (Nikon, Tokyo, Japan). The obtained cell suspensions were stained with Trypan blue, and their concentration and viability were measured using Countess II Life Technologies Cell Counter (Countess™ Invitrogen, Waltham, MA, USA).

### 2.3. Cell Culture and Establishment of Epithelial Layer

**Figure 1:**
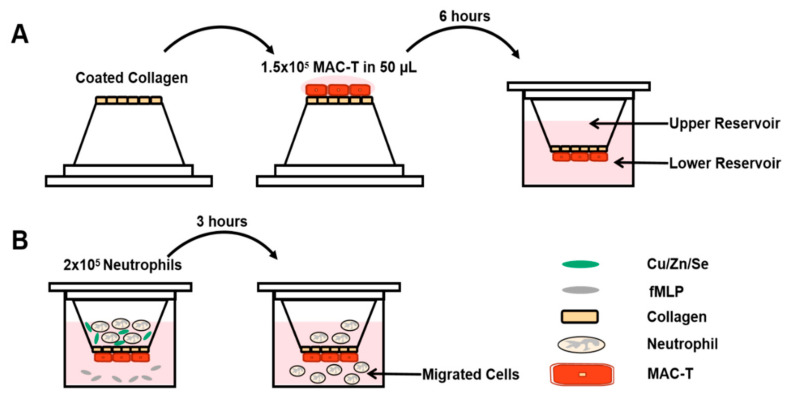
Schematic of experimental design. (**A**) MAC-T were seeded on inverted permeable supports; the supports were placed into a culture dish and incubated at 37 °C to obtain about 90% confluence. (**B**) For selected experiments, neutrophils and additional agents (e.g., Cu) were added to the upper reservoir; fMLP (100 nmol/L) in RPMI 1640 was used as the chemotactic solution in the lower reservoir. Migrated neutrophils were enumerated by the flow cytometer.

The bovine mammary epithelial cell (MAC-T) was obtained from Jilin University (Jilin, China). MAC-T cells were cultured in Dulbecco’s Modified Eagle Medium: Nutrient Mixture F-12 (DMEM/F12; Gibco, Grand Island, NY, USA) medium supplemented with 10% FBS and 1% penicillin/streptomycin (P/S; Solarbio, Beijing, China) at 37 °C in a humidified atmosphere (5% CO_2_/95% air). Cell migration assays were performed by using Transwell chambers (3.0 μm, 24-well insert; Corning, Lowell, MA, USA). Using sterile forceps, the permeable supports were inserted into a sterile 25 mm-deep tissue culture dish. Each Transwell permeable support was coated with 0.012 g/L Collagen I (Corning, Lowell, MA, USA). Then, 50 μL of the MAC-T cells suspension (1 × 10^5^ cells) was added to each permeable support. The lid was carefully placed on the dish at 37 °C with 5% CO_2_ for about 6 h. The permeable supports were placed into a 24-well plate containing 0.2 mL DMEM/F12 per well. Then, 0.1 mL DMEM/F12 was added to the upper reservoir of each permeable support and incubated at 37 °C with 5% CO_2_. The medium was replaced every 2 days. After 3 days, the permeable supports were seeded with MAC-T, and the confluence of the cells was assessed (Figure 1A). The upper reservoir was filled with 0.3 mL DMEM/F12. The cells were sufficiently confluent when the medium did not equilibrate between the upper and lower reservoirs.

### 2.4. Neutrophil Migration Assay

A stock solution of N-Formyl-methionyl-leucyl-phenylalanine (fMLP; Sigma-Aldrich, St. Louis, MO, USA) was prepared at 10 μmol/L in DMSO (Solarbio, Beijing, China). CuSO_4_·5H_2_O (Aladdin, Shanghai, China) was diluted in HBSS (Solarbio, Beijing, China) at a concentration of 1.0, 2.0, and 3.0 mg/L. ZnSO_4_·7H_2_O (Aladdin, Shanghai, China) was diluted in HBSS at a concentration of 2.0, 10 and 20 mg/L. Na_2_SeO_3_ (Aladdin, Shanghai, China) was diluted in HBSS at a concentration of 0.2, 2.0 and 4.0 mg/L. Then, 990 μL RPMI 1640 and 10 μL fMLP were added into the lower chamber of Transwell chambers, and 100 μL of cell suspension (2 × 10^5^ Cells) was added into the upper chamber. Three chambers were selected and 100 μL HBSS was added to each upper chamber as the blank control group.

To other chambers, 100 μL of different concentrations of Cu/Zn/Se dilutions were added, forming the experimental group. The Transwell chambers were placed in an incubator with 5% CO_2_ and incubated at 37 °C for 2 h (Figure 1B). The number of neutrophils that migrated to the lower chamber was measured with flow cytometry (Beckman Coulter, CA, USA). In brief, the fluid in the lower chamber was collected into the FACS tube and cells were counted using a flow cytometer and analyzed using FlowJo software (FlowJo LLC, version 10.6.0.). All experiments were repeated at least three times.

### 2.5. Statistical Analysis

All data were presented as the mean ± SEM deviation and analyzed using the GraphPad 8.0 software. One-way ANOVA and the least significant difference (LSD) test were used for parameter estimation and hypothesis testing. Significant differences were established at *p* < 0.05 or *p* < 0.01.

## 3. Results

### 3.1. Neutrophil Isolation

**Figure 2:**
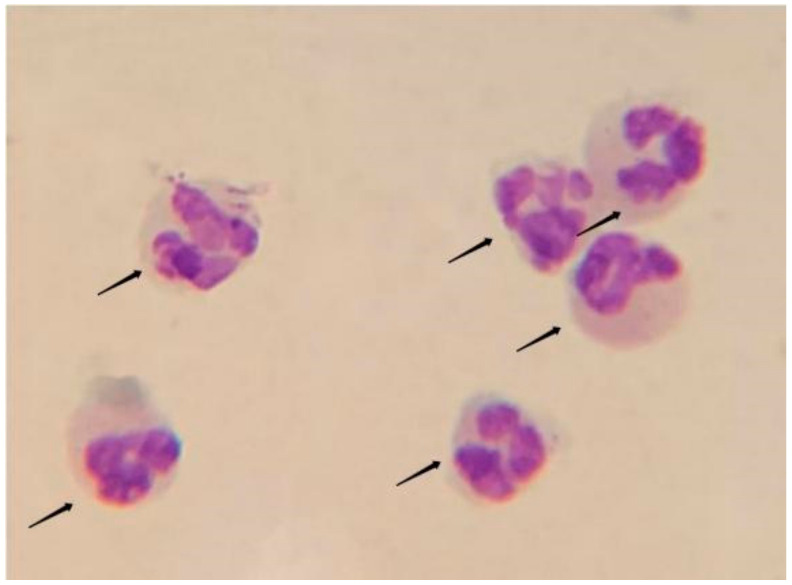
Morphology of neutrophils. Neutrophils were stained with Wright-Giemsa. Black arrows indicate neutrophil.

Neutrophils were isolated as per the manufacturer’s instructions, as described previously. The morphology of neutrophils is presented in Figure 2. The purity rate and the survival rate were both >90%, which was in accordance with the requirement for subsequent experiments and all experiments were based on this condition.

### 3.2. Effects of Cu on the Migration of Neutrophils across Mammary Epithelial Cells

**Figure 3:**
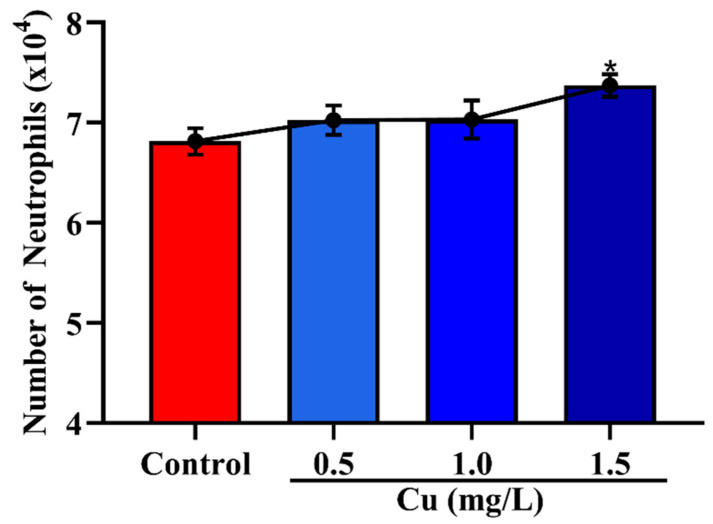
Effects of Cu on the migration of neutrophils in the trans-mammary epithelium. * *p* < 0.05 between the different concentrations of Cu (0.5, 1.0 and 1.5 mg/L) group and the control group in the same batch. Each value represents the mean ± SEM, *n* = 6.

The results of Cu on the migration of neutrophils are shown in Figure 3. The lower concentration of Cu had no significant effect on the number of neutrophils in the trans-mammary epithelium (*p* > 0.05). However, when the concentration of Cu was 1.5 mg/mL, the number of neutrophils in the trans-mammary epithelium was increased significantly (*p* < 0.05).

### 3.3. Effects of Zn on the Migration of Neutrophils across Mammary Epithelial Cells

**Figure 4:**
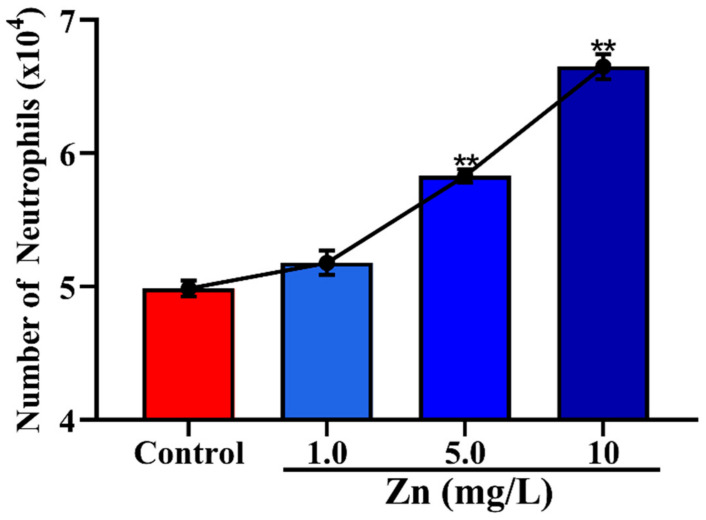
Effects of Zn on the migration of neutrophils in the trans-mammary epithelium. ** *p* < 0.01 between the different concentrations of Zn (1.0, 5.0 and 10 mg/L) group and the control group in the same batch. Each value represents the mean ± SEM, *n* = 6.

The results of Zn on the migration of neutrophils are shown in Figure 4. The results showed that Zn promoted the number of neutrophils in the trans-mammary epithelium in a dose-dependent manner. The number of neutrophils in the trans-mammary epithelium was increased extremely significantly (*p* < 0.01), when the concentration of Zn was 5.0 mg/mL.

### 3.4. Effects of Se on the Migration of Neutrophils across Mammary Epithelial Cells

**Figure 5:**
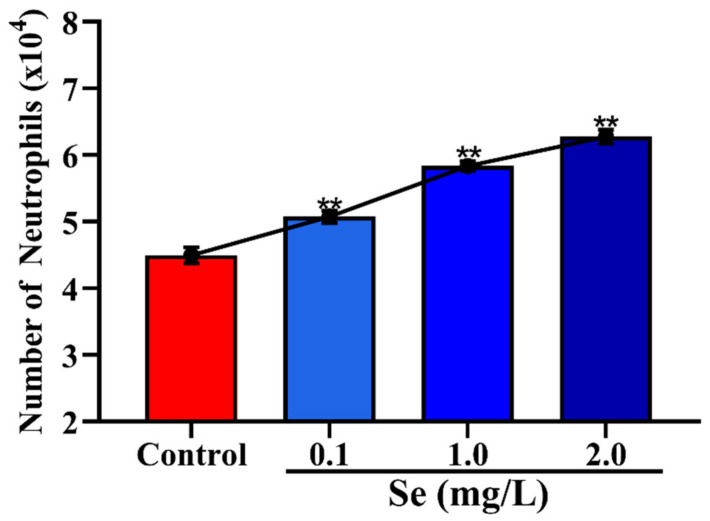
Effects of Se on the migration of neutrophils in the trans-mammary epithelium. ** *p* < 0.01 between the different concentrations of Se (0.1, 1.0 and 2.0 mg/L) group and the control group in the same batch. Each value represents the mean ± SEM, *n* = 6.

The results of Se on the migration of neutrophils are shown in Figure 5. The results showed that different concentrations of Se can promote the number of neutrophils in the trans-mammary epithelium in a dose-dependent manner. When the concentration of Se was 0.1 mg/mL, the number of neutrophils in the trans-mammary epithelium increased significantly (*p* < 0.01).

## 4. Discussion

Neutrophils represent the first line of mammary gland defense against invading pathogens by transmigration across the mammary epithelial cell barrier [5]. Admittedly, neutrophils’ infiltration of the mammary gland is critical for killing pathogens, but aberrant neutrophil accumulation into mammary gland tissue also causes damage, which is called the “double-edged sword” effect. The recruitment of neutrophils into the mammary gland is a multiple-step process and highly regulated by many proteins such as selectins, integrins, intercellular adhesion molecules (ICAM), and so on [27]. Studies have shown that dietary Cu deficiency augments neutrophil accumulation in the lung and reduces the deformability of neutrophils by promoting F-actin polymerization [28]. Cu deficiency also promoted the interactions between neutrophil and endothelial cells by increasing the expression of CD11b and ICAM-1 [29,30]. The lower and normal concentrations of Cu (0.5 and 1.0 mg/L) had no significant effect on the number of neutrophils in the trans-mammary epithelium (*p* > 0.05). With the increased concentration of Cu at 1.5 mg/L, the number of neutrophils in the trans-mammary epithelium was increased significantly (*p* < 0.05). This result might be because the Cu could promote the deformability of neutrophils. However, the normal physiological concentration of Cu is 0.7~0.9 mg/L in bovine serum and Cu excess may be even more harmful and can result in serious toxicity [18].

Zn is an essential ion for animal nutrition and development and plays an important role in cell functions, such as protein synthesis and enzymatic regulation, but excess Zn induces drastic toxicity symptoms. Past studies have repeatedly shown that immune cells are affected by free intracellular Zn and impaired cell migration in clinical and experimentally induced Zn deficiency [16,31]. In bovine serum, the normal physiological concentration of Zn is 0.7~1.3 mg/L [32]. Our findings on Zn are consistent with previous reports that supra-physiological concentrations of Zn (5 mg/L) can promote the migration of neutrophils (*p* < 0.01). One possible explanation is that Zn acts as a chemoattractant and enhances chemotaxis toward fMLP in rat neutrophils [33]. However, those findings could not be reproduced in human neutrophils. This phenomenon can be attributed to the species difference and the distinct activation states of resting and primed cells.

Se is one of the essential trace elements, with various important biological functions in mammals. It is a necessary component of numerous enzymes that are involved in redox reactions and this antioxidative function causes Se to protect immune cells from oxidative stress caused by respiratory burst. In bovine serum, the normal physiological concentration of Se is over 0.08 mg/L [18]. Innate immune cell functions have been shown to be impacted by Se levels. However, less information is available regarding Se levels and neutrophil functions. Selenoprotein K (SelK) is a transmembrane protein and is highly expressed in immune cells. Studies show that mice with SelK-knockout reduced the migration of neutrophils [17,34]. This effect is likely caused by the decreased calcium flux. In this study, we found that Se can promote the trans-epithelial migration of neutrophils (*p* < 0.01), which is characterized by an increase in Se concentration (from 0.1 to 2.0 mg/L). We hypothesize that Se may increase the intracellular calcium flux of neutrophils, which enhanced neutrophils’ random motility.

In summary, our results indicated that trace elements Cu, Zn and Se could promote the trans-mammary epithelium migration of neutrophils in vitro. These findings provide a theoretical and experimental basis for mammary gland immunity in dairy cows. Thus, we suggest that adding moderate amounts of different trace elements may improve the immune function of dairy cows.

## Data Availability

The data presented in this study are available in the manuscript.
